# Social network analysis to characterize women victims of violence

**DOI:** 10.1186/s12889-019-6797-y

**Published:** 2019-05-02

**Authors:** Michela Leone, Enrica Lapucci, Manuela De Sario, Marina Davoli, Sara Farchi, Paola Michelozzi

**Affiliations:** 1 Department of Epidemiology of Lazio Regional Health Service - ASL Roma 1, Via Cristoforo Colombo, 112, Rome, 00147 Italy; 2 Hospital network and risk management area of the Lazio Region, Via Rosa Raimondi Garibaldi 7, Rome, 00145 Italy

**Keywords:** Gender-based violence, Social network analysis, Emergency department, Patterns of diagnoses, First line screening

## Abstract

**Background:**

In Europe, it is estimated that one third of women had experienced at least one physical or sexual violence after their 15.

Taking into account the severe health consequences, the Emergency Department (ED), may offer an opportunity to recognize when an aggression is part of the spectrum of violence.

This study applies Social Network analysis (SNA) to ED data in the Lazio region with the objective to identify patterns of diagnoses, within all the ED accesses of women experiencing an aggression, that are signals for gender-based violence against women. We aim to develop a risk assessment tool for ED professionals in order to strength their ability to manage victims of violence.

**Methods:**

A cohort of 124,691 women aged 15–70 with an ED visit for aggression between 2003 and 2015 was selected and, for each woman, the ED history of diagnoses and traumas was reconstructed. SNA was applied on all these diagnoses and traumas, including also 9 specific violence diagnoses. SNA community detection algorithms and network centrality measures were used to detect diagnostic patterns more strongly associated to violence. A logistic model was developed to validate the capability of these patterns to predict the odds for a woman of having an history of violence. Model results were summed up into a risk chart.

**Results:**

Among women experiencing an aggression, SNA identified four communities representing specific violence-related patterns of diagnoses. Diagnoses having a central role in the violence network were alcohol or substance abuse, pregnancy-related conditions and psychoses. These high-risk violence related patterns accounted for at most 20% of our cohort.

The logistic model had good predictive accuracy and predictive power confirming that diagnosis patterns identified through the SNA are meaningful in the violence recognition.

**Conclusions:**

Routine ED data, analyzed using SNA, can be a first-line warning to recognize when an aggression related access is part of the spectrum of gender-based violence against women. Increasing the available number of predictors, such procedures may be proven to support ED staff in identifying early signs of violence to adequately support the victims and mitigate the harms.

**Electronic supplementary material:**

The online version of this article (10.1186/s12889-019-6797-y) contains supplementary material, which is available to authorized users.

## Background

Violence against women is a persistent global public health problem [1]. A report by the European Union Agency for Fundamental Rights, stated that one out of three women had experienced physical or sexual violence after their 15 [2]. Worldwide the most common form of gender-based violence is the abuse by intimate person [3]. In the United States, the latest data on the prevalence of intimate partner violence (IPV), collected in 2010–2012 by the National Intimate Partner and Sexual Violence Survey, indicated that over their lifetimes, 32.4% of American women experience severe physical violence from their intimate partner, with state estimates ranging from 25.4 to 42.1% (all states) [4].

In Italy, a recent survey conducted by the National Institute for Statistics on a vast sample of women (about 25,000) aged between 16 and 70 years, found that more than six million women suffered at least one episode of sexual and physical violence; the latter being more frequent (21%). Notably, about 10% of all episodes of violence occurred before age 16 [5]. Among women with a current or a previous partner, 13.6% of them had experienced physical and/or sexual violence [6]. In 2016, 115 women were killed, and the majority of them by a relative [6].

From a psychological point of view, violence is defined as an aggression behavior that is intended to cause physical or emotional harm extreme enough to require medical attention or to cause death. Thus, “all violent behavior is aggression, but most aggressions are not violence” [7].

For what concern gender-based violence against women, even in non-fatal cases, it is known to have several short and long-term sequelae. Negative health outcomes can range from direct effects such as fractures and other injuries to pregnancy-related complications, and other indirect outcomes such as chronic diseases, respiratory or gastrointestinal problems [8], that arise from prolonged stress or mental problems and impaired social functioning [8–10]. IPV affects woman’s physical and mental health, reduces sexual autonomy, and increases risk for unintended pregnancy and multiple abortions [11]. Thus, violence has long term consequences even if the violence has stopped or has been limited at a single abuse episode making the victims vulnerable for many diseases and conditions [12]. Taking into account the severe consequences triggered by the viscous cycle of violence, public health interventions, targeted to early recognized victims when they ask for medical treatment, are needed. The recognition of victims is important to offer them a path of care and support and improve their clinical and social outcomes [9].

Since ED is where most victims of violence access care for their traumas and symptoms [7], it may offer a good opportunity to recognize when an aggression related access is part of the spectrum of violence. Previous studies had shown that in settings where the medical staff have specific IPV training, the detection of violence from personal interviewing, may double and acute incidence reaches 4.8% [13].

A study conducted in Italy, in the Lazio region, [14] showed that women referring to the ED at least one time with an aggression have had an history of repeated ED accesses. Among the clinical conditions related to ED access history, the study described both direct violence-related symptoms such as contusions, abdominal or back pain, anxiety, dissociative and somatoform disorders, symptoms of the abdomen and pelvis, gynecological symptoms and a plethora of generic conditions that could be indirectly related to violence such as respiratory symptoms, unspecific complications of medical care, and other problems mainly related to pregnancy and urinary problems.

Our attempt is to identify specific patterns of diagnoses, within all the ED accesses of women experiencing an aggression, able to possibly recognize or predict a history of violence. We aim to develop a risk assessment tool to be used by the ED professionals in order to strength their ability to manage victims of violence.

In this scenario, SNA and in general a relational approach, may be an advantageous and potentially fruitful method to characterize connections among diagnoses potentially related to violence in women experienced at least one aggression episode. SNA is an extremely flexible research tool capable of mapping and measuring relationships and contact between people, groups or organizations [15]. Recently, SNA has been applied to administrative data to highlight possible unknown or infrequent interactions between poly-pharmacy and comorbidities that could be useful when improving the management of chronic diseases [16]. To our knowledge this is the first study using SNA to quantify or catch the strength of connections among different violence and other clinical circumstances using administrative data in ED setting.

## Methods

### Data collection

A cohort of 124,691 women aged 15–70 years old who sought at least one visit for aggression at an ED of the Lazio Region during the period from 2003 to 2015 was selected. Only women who were resident in the Lazio Region at the aggression event were included in the study.

For each woman, ED history was retrieved from the Healthcare Emergency Information System (HEIS) of the Lazio region. The ED history was defined as all diagnoses and traumas reported in all ED visits that occurred during the 3 years preceding the latest aggression visit.

Diagnoses were divided into violence and not violence category. The violence diagnoses category were used as a proxy of gender-based violence and it included diagnostic codes related to symptoms and signals of psychological, physical or sexual abuse: “*Adult maltreatment unspecified*” (ICD9-CM code: 995.80); “*Adult physical abuse*” (ICD9-CM code: 995.81); “*Adult emotional/psychological abuse”* (ICD9-CM code: 995.82); “*Adult sexual abuse*” (ICD9-CM code: 995.83); “*Adult neglect*” (ICD9-CM code: 995.84); “*Other adult abuse and neglect*” (ICD9-CM code: 995.85); “*History of physical abuse”* (ICD9-CM code: V15.41); “*History of emotional/psychological abuse*” (ICD9-CM code: V15.42); “*Counseling for victim of spousal and partner abuse*” (ICD9-CM code: V61.11). The presence of one of these diagnoses in the ED history identified the group of women victims of violence (WVV) among the study population. The not violence diagnoses category was made of 13 different diagnostic groups, with a previously reported association with gender-based violence (Infectious diseases; Psychoses, Alcohol or substance abuse; Mental illnesses; Neurological diseases; Circulatory diseases; Genitourinary system diseases; Respiratory diseases; Digestive system diseases; Symptoms, signs and ill-defined conditions; Pregnancy-related conditions; diagnoses where head and face were involved independently from the traumatic origin; Traumatic and musculoskeletal system and connective tissue diagnosis) and the group including all other diagnoses that have not previously reported association with gender-based violence. We also categorized ED visits in which the woman left before being examined as missing diagnosis and included them in the analysis as potentially indicative of a violent episode.

For what concern traumas, we took into account if the aggression was an isolated event or repeated (i.e. more than one ED visit for aggression). Also, we included information on the occurrence of unintentional traumas based on place of occurrence: work/school, road traffic accident, home or other place.

To sum up, we performed the analysis on ED history based on a set of 29 clinical information: 9 WVV diagnoses, 15 other diagnostic groups and 5 traumas groups (see Additional file 1).

For each woman, we also gathered, from HEIS, the available demographic information on age at aggression visit (categorized into four groups: 15–24, 25–34, 35–54, and 55–70 years), place of residence (categorized into Metropolitan area of Rome or outside) and nationality (categorized into Italian or foreign).

### Statistical methods

Selected population (124,691 women) was randomly divided into a development subsample (*n* = 93,519 corresponding respectively to 75% of the total study population) and a validation subsample (*n* = 31,172). To retain the percentage of WVV observed in the study population (0.47%) a proportional stratification was performed [17]. On development subsample a SNA approach to identify violence diagnostic patterns from the ED history was performed. Then, on the basis on SNA results a predictive model was developed to evaluate the impact of these diagnostic patterns on the odds for a woman of belonging to the WVV group in the validation subsample. Finally, we proposed a risk chart as a potential framework for ED staff for prioritizing and quickly manage health risks potentially related to gender-based violence.

All analyses were performed using R-gui software and network dedicated packages [18]. SNA was graphed using Gephi [19], an open source software platform that allows interactive exploration and analysis of complex networks.

#### Social network analysis

The SNA is a set of integrated techniques that analyzes relationships using graph theory and matrix algebra [20]. Generally, a social network is represented as a graph where nodes can represent different entities (e.g. individuals, feelings, proteins, words etc.…) and their connections represent relationships between them, valued for some relevant aspect of the relationship. In our analysis, we defined as a node each diagnoses and traumas reported in each ED visit.

To build the social network graph, we arranged information in a NxN symmetrical matrix X where the generic cell x_ij_ expressed a joint frequency. The latter is given by the number of times the node c_i_ (diagnosis or trauma *i*) was retrieved together with the node c_j_ (diagnosis or trauma *j*), namely the number of times that this couple of diagnoses was reported in ED history of women included in the development subsample.

We then normalized the matrix by reducing all nodes to the same marginal frequency using the iterative proportional fitting method (IPF). IPF method is a mathematical procedure whereby the original values are iteratively adjusted to fit row and column marginal values by leaving unchanged the relationships between nodes and the symmetry of the matrix [21, 22]. We constrained all nodes to have marginal frequencies equal to 1 [23]. Normalization was used to avoid artifactual relations from the most common ED diagnoses/traumas in the raw data.

Only the nodes that showed a strong association in the normalized matrix were evaluated. To identify strong associated nodes, we used the criteria of joint frequencies higher than the ninety-fifth percentile of the normalized joint frequencies (NJF) distribution. This threshold was chosen in order to exclude associations due only to chance.

We used the analysis of communities and centrality measures in order to identify groups of nodes more strictly jointly connected and to describe their role within a violence network.

Community within the network is defined as “a group of nodes of a graph which are more strongly connected to each other than with other nodes in the same graph” [24]. To identify a community within the network, three different clustering algorithms (Optimal, Edge betweenness, and fast greedy clustering) were performed. Modularity coefficient that indicates how well a network decomposes into communities was used to define the best clustering algorithm [25, 26].

For what concern centrality measures we used degree, closeness and betweenness in order to quantify the strength of the connection between a given diagnosis or trauma and violence diagnoses. Specifically, Degree centrality quantifies nodes with a great number of connections. Closeness centrality quantifies nodes easily reachable from other nodes based on the geodesic distance between them. Finally, Betweenness centrality quantifies nodes that frequently occur in the shortest paths between other nodes [27].

Social network graph robustness was tested comparing ten different networks building from equal sizes and stratified data subsets, obtained by randomly splitting the original dataset. The final network represented the most robust network with the only association between nodes confirmed within the ten subsets.

#### Validation of SNA results

The patterns of diagnoses generated from the final network (SNA procedure) was analyzed in multivariate logistic regression models as predictors, for a woman, of belonging to the WVV group in the validation subsample. The response variable was set up to 1 if a violence diagnosis was reported in the ED history and zero otherwise. The model was adjusted for demographic characteristics that could affect both the occurrence of diagnoses as well as the risk of being victim of violence.

In order to assess the generalizability of results, a stratified 10-fold cross-validation process was performed on the development dataset to find the optimum model. Each of the ten subsets was built to be balanced in terms of WVV cases. We measured the accuracy of the predictive models through the Receiver Operating Characteristic (ROC) curve using the Cross-Validated Area Under the ROC Curve (AUROC) [28]. The AUROC shows the trade-off between true positives (sensitivity) and false positive (1-specificity) at all possible thresholds. It is a more discriminating performance measure for accuracy [29], and is invariant to relative class distributions [30].

The coefficients and the constant terms from the predictive model were combined to obtain a risk chart that aims to be an easily graphical summary tool potential useful to ED staff in the management of women victims of violence. In particular, we first obtained a risk score summing up the coefficients and then converted it into a risk percentage using the following formula [25]:

$$ \frac{e^{\left( risk\ score\right)}}{1+{e}^{\left( risk\ score\right)}} $$

As an example, we calculated a distribution of risk percentages obtained by splitting our population according to diagnoses pattern from SNA (i.e. communities) and age and nationality of women. The distribution was then visually represented using a graduated scale of colors produced on the basis of interquartile distribution of risk percentage.

## Results

A cohort of 124,691 women experiencing an aggression (i.e. women having at least one ED visit for aggression) during the period 2003–2015 (4% of women who had at least one ED visit during the same period) was identified with a total of 400,369 visits during the three years before the aggression visit. In this cohort we identified 580 WVV women (0.47%) with at least one ED visit reporting a diagnosis code related to psychological, physical or sexual abuse. Table 1 compare the demographic and ED history characteristics of the WVV women and all the others. WVV women were younger, more likely to be foreign, and residents in the metropolitan area of Rome. Victims of violence had a higher frequency of repeated ED visits for aggression. They more frequently arrived to ED by ambulance (30% vs 15%), and 15% of the ED visits among WVV ended with the patient leaving before being diagnosed, compared with the 7.7% of all other women.

**Table 1:** Characteristics of women experiencing an aggression by presence of violence diagnosis. Lazio Region, 2003–2015

Women characteristic		WVV *N* = 580	All other women *N* = 124,111
		n (%)	n (%)
Age group	15–24 years	153 (26.38)	18,296 (14.74)
	25–34 years	192 (33.10)	31,877 (25.68)
	35–54 years	214 (36.90)	59,194 (47.69)
	55–70 years	21 (3.62)	14,744 (11.88)
Nationality	Italian	316 (54.48)	62,063 (50.01)
	Foreign	263 (45.34)	27,796 (22.40)
	Missing	1 (0.17)	34,252 (27.60)
Place of residence	Metropolitan area of Rome	361 (62.24)	64,396 (51.89)
	Outside Rome	219 (37.76)	59,715 (48.11)
Repeated Aggression	No	385 (66.40)	104,129 (83.90)
	Yes	195 (33.60)	19,984 (16.10)
Repeated Other Trauma	No	95 (16.40)	100,570 (81.10)
	Yes	485 (83.60)	23,543 (18.90)
ED history characteristics		ED visits WVV *N* = 4062	ED visits All other women *N* = 396,307
		n (%)	n (%)
Arrival	Ambulance	1228 (30.23)	60,323 (15.22)
	Self transporting	2689 (66.20)	331,290 (83.59)
	Helicopter	39 (0.96)	1343 (0.34)
	Other	106 (2.61)	3104 (0.78)
Outcome	Hospital admission	419 (10.30)	33,526 (8.48)
	Refusal hospital admission	369 (9.08)	26,700 (6.73)
	Left ED without visit	610 (15.02)	30,378 (7.66)
	Death	0 (0)	83 (0.02)
	Other	2664 (65.60)	305,620 (77.11)
Triage	Red	147 (3.62)	1587 (0.40)
	Yellow	851 (20.95)	35,488 (8.95)
	Green	2737 (67.38)	307,561 (77.61)
	White	299 (7.36)	48,259 (12.18)
	Missing	28 (0.69)	3370 (0.85)
	Not executed	0 (0)	42 (0.01)

### Social network analysis results

**Fig. 1:**
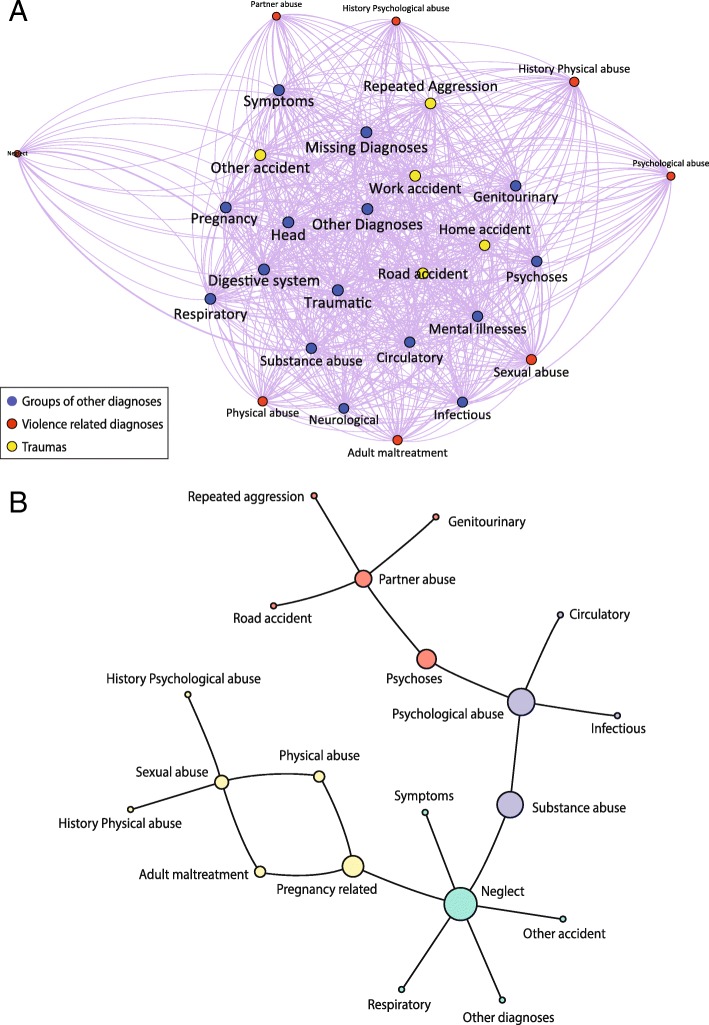
Representation of the network (part **a**) and communities (part **b**). Legend: The network represents connections between violence-related diagnoses, other ED diagnoses and traumas. The orange, violet, green and yellow communities are detected as the strongest links (over the 95th percentile) within the network

Visual inspection of the social network graph reveals a highly dense network (density equal to 0.85%), since each node is connected with the majority of the other nodes at least once (Fig. 1 part A). Each node represents a specific category of diagnosis: violence (red color) and not violence (blue color) diagnoses and trauma groups (yellow color).

Some nodes of the network were bigger than others, reflecting the greater weight of entities within the network. We can observe that the diagnoses more frequently reported during ED visits by women experiencing an aggression were: ill-defined symptoms, Traumatic and musculoskeletal system diagnoses, Head and face diagnoses, and Digestive system diseases. The trauma more frequently reported was unintentional trauma that occurred in place different from work, school, road or home. As expected, violence diagnoses showed a lower frequency of reporting and therefore were receded to the periphery of the network. Nevertheless, when we highlight the strongest associations (joint frequencies higher than the 95th percentile of the NJF distribution) in the graph, a complex group of connections with the violence diagnosis emerged (Fig. 1 part B).

We identified four communities each representing a specific patterns of diagnosis potential related to a specific violence code. The *violet* community is characterized by a psychological abuse diagnosis (adult emotional/psychological abuse) and it showed the highest connectivity level with alcohol or substance abuse, circulatory system and infectious diseases. In the *orange* community in which a partner abuse diagnosis (counseling for victim of spousal and partner abuse) is involved, the strongest links were with road traffic accident, psychosis disorders and genitourinary diseases. This community is characterized by a strong link with the repeated aggression within the ED history.

The *green* community is centered on a multi-type maltreatment diagnosis (adult neglect, adult abuse and neglect, adult maltreatment unspecified) and is strongly connected with respiratory diseases and ill-defined symptoms, other traumas or other diagnoses with not previously reported association with gender-based violence. The *yellow* community is characterized by a strong link between sexual abuse and the violence diagnoses of adult maltreatment, physical abuse and history of physical or emotional abuse. Pregnancy related conditions are the only not violence diagnosis connected within this community.

Centrality measures identified the diagnoses that play a key role in the networks (see Additional file 2). Among these diagnosis, Adult neglect could be considered the most important violence diagnosis since it showed the highest value of all centrality measures (betweenness 240; closeness 0.47). The diagnoses of alcohol and substance abuse (betweeness 176) and Pregnancy related conditions (betweeness 141) acted as bridge since they allow connections between green and violet communities the former, and between yellow and green communities the latter. Moreover, Pregnancy-related conditions played a central role (closeness 0.45) in the yellow community, whereas alcohol and substance abuse (closeness 0.33) with the psychological abuse (violet community). Within the orange community, psychoses is the diagnosis that are most strongly connected with partner abuse, and it was also a bridge with violet community, meaning that it allow a connection between two different violence pattern, i.e. partner abuse and psychological abuse.

To sum up, Among all women reporting an aggression, the SNA approach, based on clinical information from the past ED visit, identified 12 key diagnoses and traumas and 4 violence-related diagnoses patterns (i.e. communities) that could be sentinel of an hidden violence.

For instance, when a woman seeks care at ED for an aggression with an history of alcohol and substance abuse with respiratory diseases, circulatory disease, and ill-defined Symptoms, this could suggest that the aggression could be part of the spectrum of violence. In particular, SNA is suggesting that, without any other known medical reasons, this pattern could be a first line warning of a violence network to be further investigated by the ED staff.

### Validation of SNA results

Table 2 report the risk, for a woman, of belonging to the WVV group for each of the 12 key diagnoses and trauma found to be relevant in our network, along with the available demographic characteristics (Table 2). The diagnoses and traumas related to the likelihood of being a victim of violence were psychoses, alcohol or substance abuse, pregnancy-related conditions, and the other diagnosis group. Women with an history of repeated aggression had a more than double risk to be a victim of violence compared to women experiencing an isolated aggression.

**Table 2:** Results of the predictive model

Variables	OR	95%CI	*p*-value
Infectious diseases	0.80	(0.42;1.51)	0.487
Symptoms	1.06	(0.78;1.45)	0.707
Other diagnoses	1.37	(1.04;1.80)	0.024
Psychoses	2.28	(1.45;3.60)	< 0.001
Alcohol or substance abuse	2.05	(1.29;3.24)	0.002
Circulatory diseases	1.09	(0.68;1.73)	0.731
Pregnancy related condition	1.42	(1.12;1.81)	0.004
Genitourinary system diseases	1.13	(0.87;1.49)	0.360
Respiratory diseases	1.11	(0.82;1.50)	0.486
Road traffic accident	0.90	(0.69;1.17)	0.428
Other accident	1.28	(0.97;1.69)	0.082
Repeated Aggression			
No	–		
Yes	2.35	(1.90;2.90)	< 0.001
Age			
15–24	–		
25–34	0.66	(0.51;0.85)	0.001
35–54	0.45	(0.35;0.58)	< 0.001
55–70	0.18	(0.11;0.32)	< 0.001
Place of residence			
Outside Rome	–		
Inside Rome	1.70	(1.40;2.06)	< 0.001
Nationality			
Foreign	–		
Italian	0.58	(0.48;0.70)	< 0.001
Costant	0.01	(0.01;0.01)	< 0.001

Legend: Results expressed as Odds Ratio (OR) and their 95% Confidence Intervals (95%CI)

With the respect to demographic characteristics, younger women (15–24 years), foreign and women resident in the metropolitan area of Rome had the highest risk.

The model reports a good predictive accuracy and good predictive power of the risk score in the validation data set, as indicated by an AUROC of 0.72 (95%CI 0.68;0.76) when used to predict the likelihood of being a victim of violence in an independent set of patients (Additional file 3). The predictive model confirm that diagnoses patterns identified through the SNA approach are meaningful in the violence recognition.

Table 3 reproduces the coefficient risk of the predictive model in terms of percentage risk using a color scale from red, to express the highest percentage risk group, to green, the lowest.

**Table 3:**
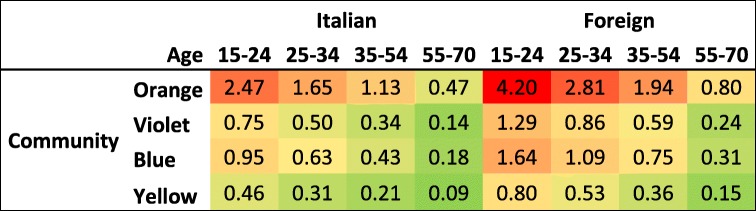
Risk chart of violence risk for Italians vs foreigners by age groups and violence communities

The combined risk coming from diagnoses included in each community represents rows, while age group and nationality were reported in columns. Looking at communities, the orange one, that includes repeated aggression and psychoses, genitourinary system disease and road traffic accident, has the highest risk, followed by the blue and violet community. The yellow community, as expected, have the lover risk since it includes only one key diagnoses, i.e. pregnancy related conditions. Coherently with the model results, the risk decreases while age increasing and is higher in foreign women, being, for example, the risk for a younger foreign woman double than an Italian peer.

The risk chart is a flexible tool to represents any scenario of diagnoses and socio-demographic characteristics that could be relevant in the recognition of violence in the ED setting.

## Discussion

At the International level, gender violence is recognized as a violation of human rights and discrimination. The worldwide high prevalence and the broad range of physical and mental health consequences make the gender-based violence a critical public health issue [9]. Evidence established that health-care providers should screen those having conditions that may be caused or complicated by violence [9]. The study of all the symptoms and conditions related to violence is crucial to correctly identify women and offer them effective care.

To our knowledge this this is the first study applying SNA in a predictive way in Italy. SNA approach allowed us to investigate in depth the complex relationship between violence diagnoses and other clinical circumstances in order to define a tool to early recognize and manage women victims of violence. By identifying the population at risk as the population of women who sought ED visit for aggression, the tool could represent a first line screening to discriminate when an aggression trauma is part of violent behavior or is an isolated event. In more detail, when a woman presents a higher-risk pattern of diagnoses she is called for a 2nd line screening to ascertain and eventually confirm the violence. Results of our analysis suggest that at most 20% of all women experiencing an aggression should be called for the 2nd line screening, since in our study population a number ranging from 580 to 25,000 women presented a violence diagnosis or a violence pattern of diagnoses at high risk. Having a first line screening in the ED setting had the advantage to lead to a better management of the victims reducing the well-known risk of the violence escalation especially in the IPV context [31]. Moreover, this tool is well suited for the Italian context where the majority of victims do not report violence nor to legal authorities nor to relatives or friends and even lower than 5% of victims ask for specialised support services (including victim counselling centres and ED staff or other medical workers). One key point to understand this undereporting is the biased perception regarding the severity of gender-based violence since the majority of women experiencing violence (about 65%) do not consider themselves as victims of a crime [5]. As a consequence, the ED could represent the first and the only contact of women victims of violence with health services.

Thanks to SNA approach we were able of creating a hypothesis-generating graphic representation of the complex phenomenon of violence, from hidden cases to occasional assault, using ED diagnoses and traumas, and their reciprocal connections to understand hidden relationships better than conventional descriptive statistical approaches without needing strong a priori assumptions. In particular, we identified some relevant pattern of diagnoses and traumas reported in the ED history that may be potential signals of a violence history.

One of the strongest signal of violence in our study was the repeated aggression retrieved by a retrospective reconstruction of all ED visits referred to the same woman. Moreover, this clinical feature is the main predictor within the pattern of diagnoses related to a potential violence by partner (orange community). This finding is coherent with the literature on domestic violence from other countries, focusing on the chronic dimension of violence which has a number of stress-related clinical outcomes such as genitourinary disease [32] or psychosomatic [33]. Traumatic event and/or chronic stress of domestic violence may cause also a wide range of psychological conditions from personality disorders (e.g. depression or anxiety) to non-specific physical symptoms (e.g. headache or musculoskeletal pain) [34, 35].

The presence of these diagnosis within a violence network can also provide useful information on the context where the violence occurs, i.e. domestic violence. Nevertheless, not all the networks are related to clinical conditions in the same strong way. In particular, we found that the community centered on sexual and physical violence (yellow community) presented only pregnancy outcomes as predictor. The predictive role of diagnoses related to complications of pregnancy is similar with what has been found in Davidov et al. [11, 36] and in the previous study on Lazio Region [14]. Pregnancy may alter the pattern of assault, with pregnant women more likely to be struck on the abdomen, which has significant consequences for pregnant women who have been found to have higher risk of obstetric complications that warrant prenatal admission [37]. It is unclear if the mechanisms causing these complications are direct or indirect; for example, is it the abdominal trauma that results in negative pregnancy outcomes, or is it that abused women do not receive prenatal care and therefore have worse health conditions [38]. The reproductive age has been recognized such a vulnerable period of women life to IPV, so that United States Preventive Services Task Force recommends to screen for IPV the entire population of fertile women [39].

Alcohol and substance abuse diagnoses, as expected from previous studies [5, 32–35], was found to be another strong predictor of violence. In fact, alcohol use in both victims and aggressors was found to be associated with more severe violence. The relationship between substance and alcohol use and domestic violence is bidirectional: while substance abuse places women at increased risk of violence, it is also true that women exposed to violence are at higher risk to abuse substances/alcohol [40]. Another important aspect of the association between violence against women and alcohol is that victims tend not to be self-sufficient, are more socially isolated, and may drink in response to the negative (and unaddressed) psychological sequelae of violence [41].

Although our study had not the objective of identifying violence victims based on their socio-demographic context, some interesting findings emerged. By looking at age, our study found a disproportionately higher risk in younger women with a decreasing risk while age increases. This trend is coherent with the age-related prevalence reduction reported in the Italian survey [42, 43]. The higher risk in the younger women is well established and seems to be most related to sexual abuse from unknown aggressor. It’s interesting to note that younger women have an higher concern about physical or sexual violence in the public domain than in the private domain, differently from older ages [2]. Foreign women represent another high-risk group in our study, with risk even twice greater than Italian women. This could be explained by underlining cultural and social factors that could affect in a different way the perception of risk as well as the severity of the violence phenomenon [32]. A previous study on mortality from domestic violence showed that women born in countries with low gender equity levels had increased risks of mortality, thus suggesting lack of empowerment as an exacerbating factor [43]. Finally, women resident in the metropolitan area of Rome seems to be more at risk compare to women residing in other parts of the region. Some interpretation could be hypothesized. Firstly, metropolitan areas are known to offer a high ED supply that could enhance the accessibility for violence victims too even because thet could choose more easily an ED away from her residency place [health care violence]. Also, metropolitan areas are characterized by a greater heterogeneity in social organizations and in collective efficacy that could be related to high crime rates, including violence behavior [44].

Some critical aspects of our study should be noted. Our study did not find some key factors related to gender-based violence. Firstly, our study failed in finding a strong connection between violence and missing diagnosis, i.e. ED visits in which the woman left before being examined, potentially indicative of violence-related medically unexplained symptoms [35]. Some authors reported lost of follow-up is critical to successfully detect other violence-related clinical outcomes [39] resulting in a possible lack of SNA capability to detect other meaningful patterns of diagnoses. Further investigation on this point is required. Secondly, another unexpected finding was the lack of strong connection between violence and head and face diagnosis. Previous research showed that battered women frequently report injuries to the upper part of the body [35] in particular skull/face fractures [36], and more in general superficial injuries and contusions [36]. The Manual of Forensic Emergency Medicine [45] states that the location of injuries may represent a defensive posture, for example, contusions, fractures or sprains to the upper extremities may be sustained while attempting to deflect blows; injuries involving both sides of the body, usually the arms and legs, are often used as markers of self-defense from violent attacks.

More generally, we cannot have excluded that some missed findings can be attributable to some conservative choices in SNA analysis. We must recognize that considering only associations with centrality measures over the ninety-fifth percentile means we may have missed some relationships that fall below this threshold. Our choice was aimed at excluding links due to chance alone. Anyhow, we performed a sensitivity analysis by comparing ten random samples derived from cross-validation to evaluate differences in graphs from the 95th percentile cutoff.

The lack of additional data that might have been relevant to understand the whole picture of violence-related consequences, such as pharmaceutical prescriptions for specific drugs (e.g. antidepressant), absences from work, other socio-economic factors were not available for this analysis.

Among future developments, we plan to validate the algorithm in clinical practice using data on a small sample of women that are victims of violence, for example those followed by some of the victims counseling centers active in our region. Emergency physicians should recognize that repeated domestic assault is common, and that previous assaults may have occurred when they evaluate a patient. Primary prevention of violence must begin early because a large percentage of victims are young.

## Conclusion

SNA has proven to be a suitable instrument to analyze ED information in our study, and to analyze a large dataset of multiple relationships among commonly reported diagnoses of victims of violence. From a dense system of relationships in which each diagnosis is often mentioned with one another, the SNA method is able to detect clear diagnostic patterns within which cross-references are more frequent, while diagnoses external to the group are rare. The method also clarified particular roles played by some diagnoses; there are some diagnoses that occur together, while others are found in different violence patterns, that connected different violence processes. The model may be proven to support ED staff in identifying early signs of violence to adequately support the victims and mitigate the harms.

## Additional files

Additional file 1: ICD9-CM codes for selected violence diagnoses, other groups of diagnoses and trauma groups. List of diagnosis and traumas used in the SNA procedure. (XLSX 13 kb)

Additional file 2: Centrality Index Values for each node. Betweeness and closeness indexes for diagnoses and traumas considered in the SNA procedure. (XLSX 9 kb)

Additional file 3: Cross-validated ROC curve for the predictive model of the risk for a woman of belonging to WVV group. Cross-validated ROC curve on the validation subsample of women. (JPG 463 kb)

## Abbreviations

95% CI: 95% Confidence intervals
AUROC: Area under the ROC curve
ED: Emergency Department
HEIS: Healthcare Emergency Information System
IPF: Iterative proportional fitting method
IPV: Intimate partner violence
NJF: Normalized joint frequencies
OR: Odds ratio
ROC: Receiver Operating Characteristic curve
SNA: Social Network Analysis
WVV: Women victims of violence
