# Supplementary figures and images for: Social network analysis to characterize women victims of violence
Source: BMC Public Health. 2019 May 2;19:494. doi: 10.1186/s12889-019-6797-y (PMC6498634; doi:10.1186/s12889-019-6797-y)

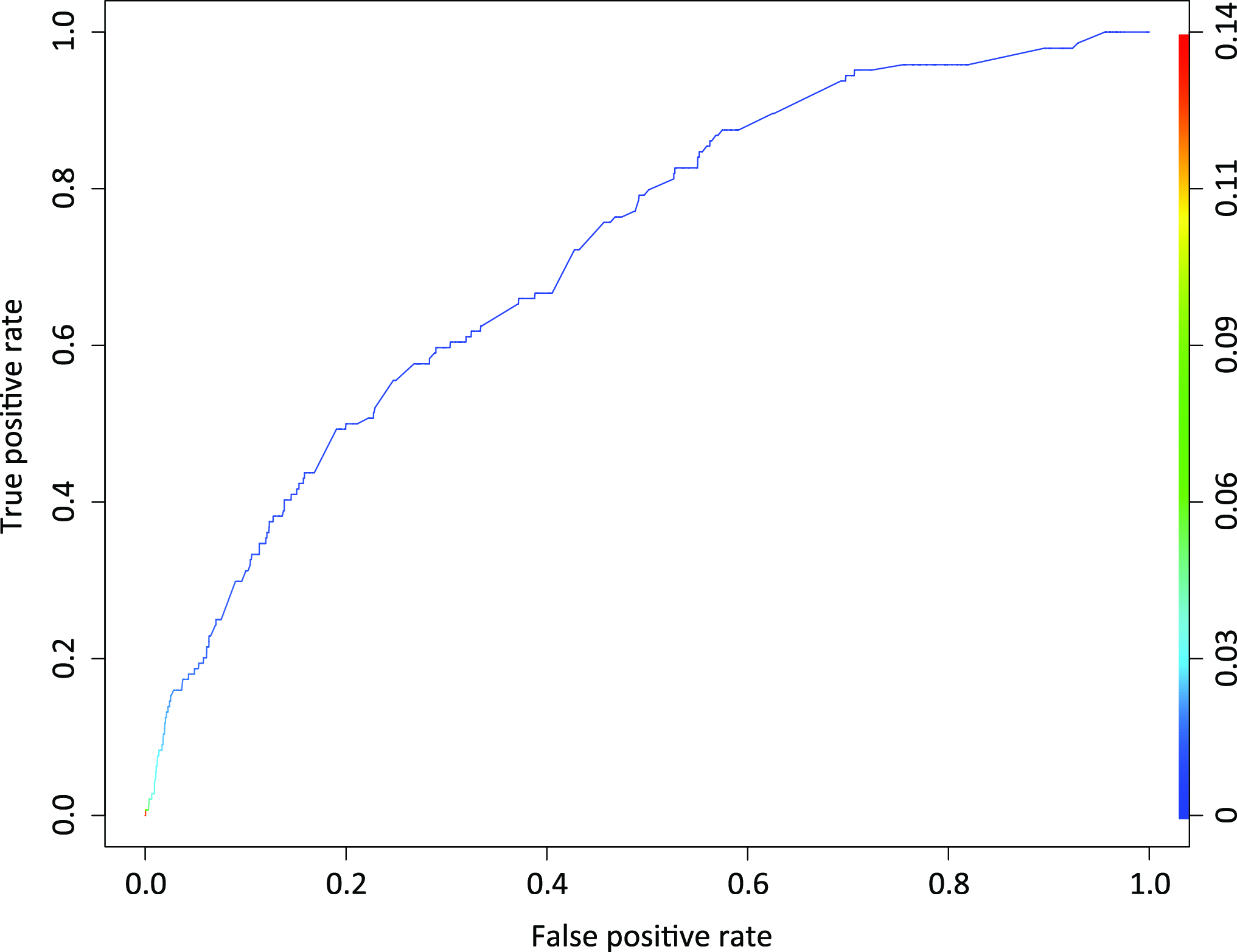

Supplement: Supplementary file 3 — Cross-validated ROC curve for the predictive model of the risk for a woman of belonging to WVV group. Cross-validated ROC curve on the validation subsample of women. (JPG 463 kb) [file 12889_2019_6797_MOESM3_ESM.jpg]
